# ‘I did what I could to earn some money and be of use’: A qualitative exploration of autistic people’s journeys to career success and fulfilment

**DOI:** 10.1177/13623613241292177

**Published:** 2024-12-20

**Authors:** Jade Davies, Rachel Melinek, Adam Livesey, Estelle Killick, Evelyn Sam, Anna Melissa Romualdez, Elizabeth Pellicano, Anna Remington

**Affiliations:** 1 University College London, UK; 2 University of Manchester, UK

**Keywords:** autism, career fulfilment, career progression, career success, employment, qualitative

## Abstract

Employment contributes to well-being, yet many autistic people who want to work face barriers to meaningful employment. Much research focuses solely on employment rates, rather than taking a more holistic view of professional trajectories and occupational experiences. We conducted semi-structured interviews with 18 autistic adults to explore their experiences of, and perspectives on, career success. Our participants highlighted the overarching importance of finding fulfilment in one’s career. Using reflexive thematic analysis, we identified five additional key themes: (1) volatile career journeys, (2) the need for continuing career support, (3) the impact (positive or negative) of social interactions, (4) the important role of organisations and (5) the catastrophic impact of poor experiences. Our findings highlight the importance of tailored, lifelong employment support and inclusive workplaces in enabling autistic people to thrive at work. Enabling autistic career fulfilment requires moving beyond entry-level hiring to foster sustainable, fulfilling employment for all autistic people.

**Lay abstract:**

Many autistic people want to work but have trouble finding jobs they like and can stick with. Most research tries to help more autistic people get jobs, but does not look at whether those jobs are fulfilling, or how people progress once they start working. We spoke to 18 autistic people about their experiences at work, and their ideas about success at work. Participants said finding fulfilment in their careers was key. We found five common ‘themes’ across the interviews. First, autistic people’s careers often take unexpected turns. For example, many participants only got diagnosed as adults, which sometimes changed their work plans. Second, autistic people might need ongoing help with their career, including help with finding jobs they would enjoy and be good at, and advice on how to progress in their job. Third, getting along with others at work is really important. Having supportive colleagues helped our participants thrive, but workplace bullying forced some to leave their jobs. Fourth, workplaces need to be welcoming to everybody. Adjustments and understanding managers helped, but many of our participants’ workplaces were not inclusive. Finally, bad work experiences can be devastating for mental health and well-being and negative experiences like bullying led some participants to quit working entirely. Our findings show that lifelong support tailored to each person and welcoming workplaces are important for autistic people to thrive at work. It is not enough to just hire autistic people – we need to help them have jobs they like and can stick with long-term.

Employment is a central part of most adults’ lives, providing not only financial security but also a sense of self-worth, social connection, meaning and purpose (Cartwright & Holmes, 2006; Duffy et al., 2016; Rosso et al., 2010). Yet, many autistic people remain unemployed, and, of those who *are* employed, many face underemployment (i.e. are in roles that require less skill, time, or capacity than they can manage) (Baldwin et al., 2014; Office for National Statistics, 2022). Efforts to address the employment disparities that autistic people face – such as research, interventions and policy campaigns – have primarily concentrated on raising employment rates. While improving employment opportunities for autistic people is undoubtedly essential, it is equally important to ensure those opportunities mark the start of a meaningful career journey. In this article, we therefore broaden the focus beyond employment figures, to examine experiences of progression, fulfilment and perceived success in the workplace.

The ability to craft careers that align with one’s interests, values, strengths and motivations is increasingly recognised as an essential component for positive outcomes in the general population (Allan et al., 2019; Duffy et al., 2016; Steger et al., 2012). Job satisfaction plays a central role in this regard (Duffy & Dik, 2013), with research showing that those who are more satisfied in their employment also gain the most benefits from being employed (Faragher, 2005; Oshio, 2021; Tatsuse et al., 2019). For some people, career progression – which includes achieving objective career-related goals (e.g. higher pay, greater status) and/or subjective career-related goals (e.g. better alignment with needs or preferences) (Guan et al., 2019; Spurk et al., 2018) – is a critical aspect of job satisfaction (Domagała et al., 2018; Sims, 2020; Stamolampros et al., 2019). Yet, disabled people may face systemic barriers in this regard. For example, disabled people typically have lower wages and poorer promotion prospects than non-disabled people (Colella & Bruyère, 2011; Deal, 2007; Kruse et al., 2018). Consequently, when disabled people are employed, they may be less likely to build meaningful careers than non-disabled people.

Building on the barriers documented for the overall disabled population, emerging research suggests autistic people face specific barriers to career progression. In our recent scoping review of 33 studies, we found that many autistic people were underemployed, despite expressing a desire to progress within their careers (Davies et al., 2024). Guided by *boundaryless career theory* (Arthur et al., 1995, 2017), we identified three key areas in which autistic people faced distinct barriers to career progression: (1) knowing-why (the ability to reflect on one’s values, skills and motivations and plan accordingly); (2) knowing-how (possessing the human capital required to progress) and (3) knowing-whom (possessing the social capital required to progress). Regarding ‘knowing-why’, studies with non-autistic people have suggested that they often perceive autistic people as experiencing difficulties with setting appropriate career goals (e.g. expressing a desire for job roles above their current skill level) (Braudis, 2017; Wong et al., 2020). In the review, we suggest these perceptions may reflect the low expectations and low presumptions of competence of autistic people. In contrast, in firsthand accounts, autistic people speak eloquently about their ambitions and future goals (see, for example, research on ‘citizen-workers’ by Nouf-Latif et al., 2019). Second, disproportionate barriers to gaining career-related skills and experience (‘knowing-how’) may translate into obstacles to career progression for autistic people. For example, several of the included studies highlighted how gaps in education or employment histories limited progression opportunities (Anderson et al., 2021; Sang et al., 2022; Sharpe et al., 2022). Third, autistic people may face specific barriers to developing the social capital (‘knowing-whom’) that may be required to progress. For example, studies show that disclosure of an autism diagnosis risks discrimination and thus fewer opportunities to progress (Buckley et al., 2021; Djela, 2021; Harvery et al., 2021; Hedley et al., 2021; Romualdez, 2021; Wood & Happé, 2021). Similarly, differences in communication between autistic and non-autistic colleagues might preclude autistic people from successfully networking and/or reaching managerial positions (Braudis, 2017; Buckley et al., 2021; Cockayne, 2019). Finally, part-time or precarious employment, driven by social-welfare systems and a lack of long-term employment support, may restrict progression (Balubaid, 2017; Berman, 2022; Hayward et al., 2019b; Lindstrom et al., 2014).

While the findings outlined above demonstrate some of the key barriers autistic people may face in achieving career progression, there remains minimal firsthand insight into how autistic people define and pursue career success (Lounds Taylor, 2017; Pellicano, Fatima, et al., 2022). This study addresses this gap to answer the question: ‘What are autistic people’s journeys to career success?’ In so doing, we draw on Life Course Theory (Elder et al., 2003), which seeks to acknowledge the impact one’s personal circumstances (e.g. their historical, cultural and socioeconomic context) have on their life trajectory (Elder et al., 2003; Elder, 1974, 1994). Central to Life Course Theory are the concepts of transitions (discrete changes or milestones), trajectories (sequences of linked changes over time) and turning points (pivotal changes redirecting life trajectories) (Hutchison, 2011). The theory also emphasises the interdependence of different life trajectories (e.g. personal, family, work and health trajectories) (Hutchison, 2011).

## Method

### Community involvement

We adopted a participatory approach, with both autistic and non-autistic team members contributing to the research design and delivery. Building on previous work, non-autistic researchers generated the research topic, and invited autistic researchers and self-advocates to be involved in making decisions about the remainder of the project. For example, autistic authors decided on the data collection technique (semi-structured interviews) and designed study materials (e.g. emails, information sheets, consent forms, interview guide) to ensure they were accessibly written and sensitive to participants’ needs and preferences. Two autistic authors did not have a formal background in qualitative research, so they were trained in interview techniques and reflexive thematic analysis. Both autistic authors, with support from a non-autistic author, conducted interviews with participants, which we felt helped to foster a sense of shared understanding and improve rapport building (Pellicano, Lawson, et al., 2022). One autistic author worked with non-autistic authors to analyse the data, using their lived experiences of trying to navigate employment as an autistic person to provide more relevant and nuanced interpretations of the data. The findings were drafted by a non-autistic author and presented to autistic authors for feedback and input regarding the implications of the findings for future research and practice. The autistic authors provided crucial insights in this regard. For example, they emphasised the apparent importance of *career fulfilment*, as well as the importance of recognising intersectionality in autism-employment research and practice.

### Participants

Eighteen autistic adults, based in the United Kingdom, took part in the study. Participants were recruited via the Autistica Network (*n* = 14, 77.8%), the Discover Autism Research and Employment (DARE) database (*n* = 3; 16.7%) and a personal contact (*n* = 1; 5.6%). The recruitment advertisement invited autistic people (including formally diagnosed and self-identified) that lived in the United Kingdom^1^ and had at least a total of 5 years of employment experience including breaks (to facilitate reflection on their career trajectory) to take part. There are growing concerns in the field regarding fraudulent research participation, especially where financial incentives are offered (Pellicano et al., 2024). To mitigate the risk of scammer participants, we did not to advertise our research via social media, instead opting for avenues specifically for autistic people. We were vigilant for published signs of fraudulent participation throughout the study process (e.g. unusual email patterns, inconsistencies in participants’ accounts, or vague responses about autistic experiences) (Pellicano et al., 2024). We carefully monitored our interactions and data collection processes and did not observe any patterns that might indicate fraudulent participation.

Of the 18 autistic people who took part, most were from a white ethnic background (*n* = 14, 77.8%) and half identified as women (*n* = 9, 50.0%) (see Table 1). On average, participants were aged 47 years (SD = 9.95, range = 34 years–64 years). Most had a formal autism diagnosis (*n* = 16, 88.9%), and almost all of those received their diagnosis in adulthood (*n* = 15 of 16, 93.8%; M age of diagnosis = 39 years, SD = 12.95). All participants reported co-occurring conditions, most commonly anxiety, unique sensory processing and/or depression (see Table 2). No participant reported having a co-occurring intellectual disability.

**Table 1.** Participant characteristics (*n* = 18).

	*N* (%)
**Gender identity**	
Woman	9 (50.0)
Man	7 (38.9)
Non-binary	2 (11.1)
**Age M (SD)**	47.28 (9.95)
**Ethnicity**	
White	14 (77.8)
Asian	1 (5.6)
Black	1 (5.6)
Mixed	1 (5.6)
Other	1 (5.6)
**Geographical location**	
London	6 (33.3)
Yorkshire & the Humber	4 (22.2)
East of England	3 (16.7)
East Midlands	1 (5.6)
North East of England	1 (5.6)
Scotland	1 (5.6)
South East of England	1 (5.6)
Wales	1 (5.6)
**Highest level of education**	
Doctorate	1 (5.6)
Master’s degree (e.g. MA, MEd, MSc)	4 (22.2)
Post-graduate education (e.g. PGCert, PGDip, PGCE)	3 (16.7)
Bachelor’s degree (e.g. BA, BEd, BSc)	3 (16.7)
Foundation degree	1 (5.6)
Vocational qualification (e.g. GNVQ, HND, BTEC)	3 (16.7)
A/AS-Level^a^	2 (11.1)
GCSEs^b^	1 (5.6)
**Number of previous employers (including current employer, if relevant)**	
7+ employers	10 (55.6)
5–6 employers	3 (16.7)
3–4 employers	5 (27.8)
**Current employment status**	
Employed full-time	6 (33.3)
Employed part-time	4 (22.2)
Employed casually	1 (5.6)
Self-employed (e.g. freelancer, sole trader)	1 (5.6)
Currently on maternity leave	1 (5.6)
Unemployed – not looking for work	4 (22.2)
Unemployed – due to health condition	1 (5.6)
**Employment sector** ^c^	
Education	6 (46.2)
Healthcare	2 (15.4)
Accountancy	1 (7.7)
Charity	1 (7.7)
Finance	1 (7.7)
Public sector	1 (7.7)
Science	1 (7.7)
**Managerial responsibilities in current role** ^c^	
No, I don’t have any managerial responsibilities	8 (61.5)
Yes, I manage processes	2 (15.4)
Yes, I manage people and processes	2 (15.4)
Yes, I manage people	1 (7.7)
**Current income** ^c^	
<£10,000	2 (15.4)
£10,000–£19,999	4 (30.8)
£20,000–£29,999	2 (15.4)
£30,000–£39,999	1 (7.7)
£40.000–£49,999	1 (7.7)
£50,000–£59,999	2 (15.4)
£100,000–£149,999	1 (7.7)
**Satisfaction with current employment M (SD)** ^c^	3.38 (1.19)
1 – Very dissatisfied	1 (7.7)
2 – Dissatisfied	2 (15.4)
3 – Neither satisfied nor dissatisfied	3 (23.1)
4 – Satisfied	5 (38.5)
5 – Very satisfied	2 (15.4)

a A/AS Levels are qualifications in the United Kingdom, typically taken between 16 and 18 years of age.

b GCSEs are qualifications in the United Kingdom, typically taken between 14 and 16 years of age.

c *n* *=* *13*; note, these questions were only presented to participants who indicated they were employed.

**Table 2.** Participant reported co-occurring conditions.

	Applies to me				
	Formally diagnosed *N*	Self-identified *N*	Not specified *N*	Total *N* (%)	Mean age (in years) of formal/self-diagnosis (SD)^a^
Anxiety	13	1	1	15 (83.3)	36 (12)
Unique sensory processing	8	4	1	13 (72.2)	43 (16)
Depression	10	1	1	12 (66.7)	30 (8)
ADHD	5	5	0	10 (55.6)	41 (14)
Alexithymia	3	6	0	9 (50.0)	47 (12)
Post-traumatic stress disorder	6	3	0	9 (50.0)	44 (14)
Physical health condition/disability	5	0	0	5 (27.8)	39 (3)
Dyspraxia	2	2	0	4 (22.2)	42 (11)
Dyslexia	2	1	0	3 (16.7)	49
Dyscalculia	0	2	0	2 (11.1)	34 (6)
Prosopagnosia	1	1	0	2 (11.1)	55 (4)
Aphasia	0	1	0	1 (5.6)	N/A
Eating disorder	0	1	0	1 (5.6)	24
Obsessive compulsive disorder	1	0	0	1 (5.6)	24
Personality disorder	1	0	0	1 (5.6)	33
Schizophrenia	1	0	0	1 (5.6)	N/A
Other^b^	N/A	N/A	N/A	6 (33.3)	N/A

a Not all participants completed this question. As such, the mean and standard deviation (SD) values presented here may be based on fewer participants than indicated the condition applied to them.

b Participants were able to indicate other factors/diagnoses impacting their employment in a free-text response box. Responses included conditions such as chronic fatigue, fibromyalgia, Irlen syndrome, asthma and hypothyroidism.

Over half of the participants (*n* = 10, 55.6%) had seven or more previous employers, and most were currently employed (*n* = 13, 72.2%, including one participant on maternity leave). A range of employment sectors were represented, with education (*n* = 6 of 13, 46.2%) being the most common. Most participants did not have any managerial responsibilities in their current role (*n* = 8 of 13, 61.5%), and almost two-thirds (*n* = 8 of 13, 61.5%) earned £30,000 or less per year.^2^ Satisfaction with current employment ranged from very dissatisfied (1) to very satisfied (5), with responses being neutral on average (M = 3.38, SD = 1.19).

### Materials

First, participants completed a demographics questionnaire, hosted on Qualtrics (2023). The questionnaire included questions regarding participants’ age, gender identity, ethnicity, highest level of education and geographical location. Participants were also asked to indicate any (formal or self) diagnoses they had, and at what age they received those diagnoses. Finally, participants answered a series of questions concerning their employment (e.g. number of previous employers, current employment status, sector of current employment, the nature of any managerial responsibilities they currently have, current salary, and satisfaction with current employment). Next, participants were invited to complete a career timeline, containing general information about their education, training, and employment history. These timelines were used as prompts by both the researchers and the participants during the interviews.

We developed a semi-structured interview schedule (Appendix 1) guided by Life Course Theory (Elder et al., 1974, 1994, 2003). We started the interviews by asking participants to reflect on the context in which they grew up. For example, prompting them to discuss the area they lived in, the decade they grew up in, who they lived with, what their parents/carers did for work, their family’s financial security, and the role models they had. Next, the interviewer asked about the participant’s educational experiences (aged 16 years and above), prompting for information about the support available, and how they made decisions about the next steps in their career. Participants were then asked to talk through their career timelines. Finally, participants were asked to reflect on their satisfaction with their career, with reference to career progression and perceived success.

### Procedure

We obtained ethical approval via the Research Ethics Committee at IOE, UCL’s Faculty of Education and Society (REC1615). All participants gave informed consent to take part. Interviews were conducted remotely via video (*n* = 15, 83.3%) or voice call (*n* = 3, 16.7%) by J.D., R.M. or A.L. Interviews ranged between 24 and 156 min (M = 83 min), reflecting differences in individual career trajectories. With prior consent, we recorded the interviews. Transcripts were then automatically generated using the built-in features of the video conferencing platforms used (either Zoom or Microsoft Teams). The first author (J.D.) reviewed and corrected each transcript for accuracy. Participants received a £20 voucher following their participation.

### Data analysis

We analysed interview transcripts using reflexive thematic analysis within a contextualist framework (i.e. reporting the experiences, meanings and realities of the participants, while acknowledging the complex ways in which such realities are constructed) (Braun & Clarke, 2006, 2019, 2021). Our analysis was primarily inductive in nature, to prioritise our participants’ lived experiences. The first author (J.D.) led the analysis, with support from three autistic and non-autistic authors (R.M., E.S. and E.K.). J.D. analysed all transcripts, while R.M., E.S. and E.K. each reviewed a subset. The authors familiarised themselves with the data through the interview and transcription processes, and/or by reading and re-reading each transcript. Data perceived as being salient were assigned preliminary codes, summarising the semantic and latent meanings within the data. The four authors met on several occasions to discuss their interpretations of the data. The use of multiple coders was not to reach a consensus, but instead to utilise the different (lived and research) experiences within the team, thus achieving a richer interpretation of meaning (Byrne, 2022). When all transcripts had been coded, the four authors met to develop, refine and name the preliminary themes, using Google Jamboard. At this stage, we also drew on Life Course Theory (Elder et al., 1974, 1994, 2003) as a deductive framework to identify career transitions, trajectories and turning points. Once the preliminary themes had been decided, two of the authors (J.D. and R.M.) involved in the analysis met with the wider team to present the findings and discuss their potential implications for future research and practice. Minor adjustments were made to the structure of the findings at this stage, such as adding sub-themes to improve readability. J.D. wrote up the results and all authors reviewed and agreed upon the final set of themes.

### Positionality

As previously outlined, this research was conducted by a team of autistic and non-autistic researchers, who all contributed to the research design and delivery. The authors also have varied experience and expertise (e.g. lived-experience, junior research experience, senior research experience). Our analysis and interpretation of the findings were influenced by this varied experience and expertise, as well as our commitment to a neurodiversity approach to research and practice (Chapman & Botha, 2023; Pellicano & Houting, 2022).

## Results

**Figure 1.**
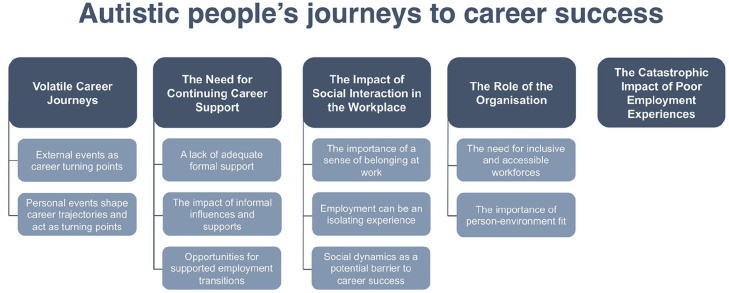
Thematic map of autistic people’s journeys to career success.

Our participants emphasised that career success – and the path towards it – was nuanced and depended on what such success meant for them. Some participants spoke of a desire for more extrinsic forms of career success, such as earning more money, and moving into higher-status positions (‘I’m definitely ready to (move) up to the next corporate level’, p. 13), while others sought more intrinsic forms of career success. For example, participants described the need to ‘help other people’ (p. 12), ‘keep learning [and] being better at what I do’ (p. 03) and feel valued and appreciated at work: ‘I want job satisfaction, feeling valued is more important to me than any amount of money’ (p. 14). Common across all participants, however, was a desire to find fulfilment in one’s career, with many perceiving ‘fulfilment’ as synonymous to, and an integral aspect of, career success. We also identified several areas of overlap regarding participants’ paths to success, which clustered around five themes (see Figure 1).

### Theme 1: volatile career journeys

#### Sub-theme 1: external events as career turning points

Participants spoke about the many twists and turns they experienced throughout their careers and identified several events which acted as turning points. For example, some participants discussed their poor educational experiences and how such experiences hindered their career prospects and employment opportunities:

In the era I grew up in which was 70s/80s, autistic children were treated as naughty children, and I had a lot of experience of punishment because of that which damaged my self-esteem and decisions were made about me that affected my possibilities of a career in the future. (p. 14)

Other events, such as widescale economic changes, also resulted in a complete change in career plans: ‘*[I was self-employed] until 2008 when the big crash happened [and] all the work dried up*’ (p. 12).

#### Sub-theme 2: personal events shape career trajectories and act as turning points

Participants also spoke more broadly about how their personal circumstances shaped their career trajectory. For example, some participants, particularly women, discussed the impact of starting a family and/or looking after their children: ‘*I had to go back part-time because the childcare costed a fortune*’ (p. 09). In some cases, having a family necessitated a complete change in women’s career:

Most people were forced out [of employment] at 28 weeks pregnant . . . and [were] back within 3 months of the baby’s birth. But I just could not even begin to contemplate doing that. So, in the end I just said, okay, fine we will just have our family. [But] I needed to earn something . . . so, I thought, oh, I’ll do some of that childminding. (p. 17)

Participants also discussed how ill-health – either their own or a family member’s – changed their plans. For example, one participant explained, ‘My dad was diagnosed with a form of cancer which impacted my A-Level results and then impacted which university I went to’ (p. 13). Other participants explained how familial ill-health led them to turn down promotions (‘my boss [offered a promotion and] I said to be honest, we’re just going through fertility testing, one of my wife’s relatives is seriously ill . . . there is quite a lot going on, so I’d rather not’, p. 11) or changed their career path entirely:

My son was going through [some health issues]. He had so many hospital appointments and in these appointments, I felt a bit lost because I didn’t know what was expected of me, and he was losing out on treatment because of me. So, I thought, I’m going to become a nurse, so I know how to help my son. (p. 04)

Most commonly, receiving an autism diagnosis or identifying as autistic was perceived as a turning point in people’s careers. Indeed, participants spoke of having to ‘completely realign [their] sense of self’ (p. 17) and ‘have lots of time off work to go through therapy’ (p. 12) post-diagnosis. For one participant who received their diagnosis following difficulties at work, the period post-diagnosis was a ‘grieving process for my career and for me, because I didn’t know who I was’ (p. 09). When they had come to terms with their diagnosis, some participants moved into different industries and/or moved into employment centred around supporting and/or working with autistic people: ‘the only other interest I’ve really got that could make some kind of career out of is autism. I thought I’ve read so much about it . . . so that was what I started doing’ (p. 09).

### Theme 2: the need for continuing career support

#### Sub-theme 1: a lack of adequate formal support

Some participants spoke of an inherent ‘knowing’ of what they wanted to do from an early age and did not feel they needed any formal career guidance or support: ‘I always knew I wanted to be some kind of scientist. That sense was innate within me’ (p. 03). Many, however, reported feeling out of their depth when it came to planning for their future. Indeed, participants highlighted that career guidance for youth was often generic and inadequate, especially for those who were perceived to be succeeding academically: ‘the background assumption was always [participant] will be all right, because you know he’s clever, he’s articulate’ (p. 15). Yet, career guidance was also felt to be inaccessible by those whose backgrounds may have resulted in fewer academic opportunities:

I didn’t feel like I could do [the jobs they were talking about]. I suppose it is a little bit, self, like a self-worth thing because I lived in a council flat, and I went to a school where a lot of people [were] middle class and had two parents. [They] had cars and my dad got drunk every day, and I had no carpet, no wallpaper, it was mouldy, my ceilings were falling out. (p. 02)

Some participants sought professional support later in their careers. Yet they felt that providers of such support did not match the suitability of the job to the participant:

All the employment schemes were based around just pushing you into a job as soon as possible so they got their bonus. They were not interested in looking at the suitability of the job, or whether it would harm you. (p. 14)

#### Sub-theme 2: the impact of informal influences and supports

In the absence of formal careers guidance, some participants reported copying their peers (‘I didn’t know what to do. . .I just asked what everyone else [was] doing’, p. 04), while others were guided by parental expectations. To that end, some participants were encouraged to aim for employment in well-respected, high-paid industries (‘my parental expectations, or my interpretation of my parental expectations, was [to] do something a bit more concrete’, p. 12), while others were encouraged to find any job that paid: ‘[I was told] you just want to get out there and get a job and earn money’ (p. 09). Participants reported how these early experiences shaped their approach to employment: ‘it was really like feast or famine in my house, we would have money for food, and then we wouldn’t . . . all I wanted to do was grow up and make money, so I could have some stability’ (p. 05).

Participants also noted the influence of the temporal and cultural context they grew up in. For example, one female participant noted, ‘My secondary school years were spent throughout the seventies in an all-girls school which was set up to turn out “young ladies”. Lots of learning to type, and things like that: “You’re all going to go and be secretaries”’ (p. 04). Similarly, participants highlighted the lack of autism awareness during their youth and suggested that their approach to career planning may have been different, had there been more awareness: ‘if I had had more information [about autism] when I was in my teens . . . it would have absolutely affected my choice of career path’ (p. 10).

#### Sub-theme 3: opportunities for supported employment transitions

Many participants reported that they would have benefitted from a more gradual, supported transition into work. Some participants suggested the utility of early part-time work as a useful tool for preparing for employment: ‘I really benefited from doing a customer service role early in my career . . . it enabled me to learn what my work persona was, and to craft that work persona in a safe environment’ (p. 13). Yet, others felt they needed more formal ‘coaching and scaffolding’ (p. 15), not just at the start of their career, but throughout. When participants did have such support, outcomes were generally positive. For example, in one case, a participant had an informal advocate who encouraged them to apply for a higher-paid position and supported them through the application and interview process, ultimately supporting them in achieving their career goals. Indeed, the participant explained: ‘without that receptionist having chosen to leave, and without [the advocate] having known that, and saying you are going to apply for this job, I wouldn’t have ended up doing that’ (p. 13). In several cases, family and friends provided similar support by identifying possible job opportunities and advocating for participants: ‘It was a friend of my mums who got me the job . . . I think they just wanted to give me that chance because of my mum’ (p. 01).

### Theme 3: the impact of social connections in the workplace

#### Sub-theme 1: the importance of a sense of belonging at work

Many participants had relatively social jobs and some actively sought a sense of community and belonging at work. Indeed, for some participants, work was the main context in which they connected with others, making work an important place for structured socialisation: ‘I like socialising, but it is difficult, and the idea of the bar work was the job gave the subject to talk about. So, I had the means to meet my social need without that same level of difficulty’ (p. 14). Positive employment experiences were often when participants felt integrated into supportive teams and developed good working relationships with their colleagues and employers: ‘That’s what’s kept me there . . . the fact that people are so nice’ (p. 03). To foster positive connections and a sense of belonging in the workplace, one participant highlighted the importance of employee resource groups: ‘one thing autistic people should 100% be doing is joining [employee resource groups] and connecting up with other neurodivergent people [because] that peer support has been so valuable’ (p. 13).

#### Sub-theme 2: employment can be an isolating experience

Despite the apparent importance of social connection at work, many participants reported experiencing interpersonal issues, which negatively impacted their experiences. For example, some participants spoke of being exploited (‘we’re [autistic people] so vulnerable to being exploited’, p. 18) and/or bullied: ‘I can identify 5 work settings where I was bullied. [Maybe] being autistic made me seem like a good choice of target for the bullies. All [instances of bullying] led to me leaving’. (p. 11). Many participants shared similar stories of their experiences of workplace conflict, which often resulted in termination, and, sometimes, employment tribunals.

Even those who did not have such severe experiences discussed social challenges at work. Often, such challenges centred around socialisation within the office environment (‘I had a supervision with the guy who was in charge of the office, and he just said you don’t fit in, you don’t talk to people. . .your attitude is really cavalier’, p. 11) and challenges navigating common workplace practices:

They do this thing at work where they want you to bring food in and share, and all have a lovely lunch. Oh, my gosh! I literally plan flu for those days, because it’s the worst, I can’t bear it. (p. 18)

Such experiences often resulted in participants masking their autistic identity (‘I was masking to within an inch of my life’, p. 09), which was not always sustainable: ‘I just reached the point where I just couldn’t cope. I just couldn’t keep up this, this mask’ (p. 08). Indeed, many participants spoke of the need to take ‘a break mentally from the [work] environment’ (p. 02), which often manifested in avoiding social work events, something many colleagues failed to understand: ‘[people think] it’s weird if someone doesn’t eat their lunch in the hall, [or] if someone never shows up in the pub’ (p. 01).

#### Sub-theme 3: social dynamics as a potential barrier to career success

Social misunderstandings were felt to negatively impact career trajectories. Some participants felt their difficulties with networking held them back: ‘I didn’t really then take part in all the sort of networking things that would have progressed my career, and that’s something that’s had an impact all the way through’ (p. 12). Similarly, participants suggested that promotions were often awarded based on social interaction, which disadvantaged autistic employees: ‘Why am I working harder than this person here, and that person there is getting all the perks and I’m getting all the shitty jobs? [It’s because] I’m not sucking up to them’ (p. 05).

Some higher-paid positions were perceived as unattainable as they often required elements of managing others, something many participants had no interest in doing (‘I’ve always wanted to be in the background, I’m totally comfortable there’, p. 18), or felt unable to do: ‘I’m never going to be a manager of people and going up to the job ranks. I don’t have those abilities’ (p. 10). Some participants who had taken on managerial positions, often before knowing they were autistic, reflected that such positions were challenging despite their technical abilities: ‘I found it a nightmare . . . I was put in these roles because I was good at the technical side, not human management . . . I get really tired of that, and I can only do it for so long’ (p. 12). Yet, social dynamics were not universally viewed as a barrier to career progression and success. Some participants highlighted autistic traits like being direct, empathetic, and avoiding office politics, which made them well-suited to leadership: ‘I’m very straight talking. . .I don’t need to be everybody’s mate, so I suppose that helps’ (p. 02).

### Theme 4: the role of the organisation

#### Sub-theme 1: the need for inclusive and accessible workforces

When considering the longevity of careers, participants often spoke of the importance of the organisation’s overall approach to inclusivity and accessibility. Unfortunately, employers were generally perceived to lack knowledge and awareness of autism, including the unique strengths autistic people might have, as well as how being autistic may impact employment experiences: ‘There are a lot of employers who have very wrong, very outdated ideas about what autism is . . . [yet], simply recognising that someone is autistic has the potential to drastically change some outcomes in employment situations’ (p. 11). In many cases, this resulted in inadequate work environments and non-inclusive cultures. Often, participants felt this started at the recruitment stage, with unclear job advertisements (‘I would look at job adverts, it was all just like just horrifying to me and I was reading the job descriptions and it was like, well I’m clearly not qualified for really anything’, p. 15) and an overreliance on job interviews which disadvantaged autistic candidates and limited opportunities: ‘I must have interviewed terribly badly. Because I’ve got quite good exam [results] which is probably more than some of the people that you’re appointing’ (p. 11). Such experiences also extended beyond the recruitment process with one participant explaining: ‘the career development process does not take into account my wider talents, but also my difficulties . . . I can’t apply to every development program, because every time I get a rejection, I take it so personally’ (p. 10).

#### Sub-theme 2: the importance of person–environment fit

More broadly, participants spoke of the sensory environment at work as being a barrier to career success, particularly within more manual, trades industries: ‘it’s like I’ve been nettled everywhere inside the whole time I’m at work. It’s so uncomfortable. The uniform, the pants, the work, the dust, the tools. It’s just torture’ (p. 02). Indeed, some participants reflected that they may have chosen more sensory-friendly workplaces had they known they were autistic earlier: ‘I probably took some jobs that weren’t suited to me, like a receptionist job. I used to work on reception, and [now] I’m like ‘how the heck?’’ (p. 05). Nonetheless, several participants had been able to sustain meaningful employment with the support of workplace adjustments: ‘The only success [I had] was working for my friend at their shop. That’s because they were willing to make reasonable adjustments’ (p. 14). Yet, some adjustments came at a cost, with one person explaining: ‘I only work part-time because I think that’s more manageable. Obviously, that means that I’m then more vulnerable financially because I don’t earn as much’ (p. 06). Furthermore, many experienced significant challenges in having their adjustments implemented: ‘[I asked] if I could have a one-to-one with somebody else just to talk over [the tasks, but] they said they can’t accommodate that’ (p. 04). As a result, some participants felt they had to turn to self-employment to curate an appropriate work environment for them: ‘the only way I could see forward is self-employment’ (p. 14). Yet, self-employment also came with its own challenges: ‘I’m not a salesperson. I can’t push anything on anyone, I’ve never ever been good at that. So, I was just like left, I had no customers’ (p. 05).

Nonetheless, where jobs, and workplaces more broadly, were perceived to be well-matched to people’s needs and preferences, experiences were thought to be more positive. For example, one person explained: ‘McDonald’s wasn’t for me, but for others, that’s brilliant, because you don’t have to talk to anybody, you stay at your station. . .you might get satisfaction from making the best fillet-o-fish ever. It’s [about] finding what makes people happy’ (p. 18). Similarly, some participants suggested using temporary work as a trial to see if workplaces would be suitable for them: ‘I chose the temping [temporary work] because I could see if I would fit into the workplace, and I could see if the job was for me’ (p. 05). Overall, many participants felt employers must do more to ensure autistic people would fit into their workplace. One person, for example, called for more employers to ‘recognise [autistic] people’s strengths and support them, because it’s ultimately when people are allowed to deploy their strengths that they flourish and are better able to manage their vulnerabilities’ (p. 03).

### Theme 5: the catastrophic impact of poor employment experiences

In many cases, the perceived absence of adequate career support and inclusive, accessible workplace environments had a catastrophic impact on participants’ mental health and well-being. Indeed, many participants spoke of the toll their negative experiences had: ‘[work is] the thing that’s kind of pushed me, in a sense, over the precipice into being seriously unwell . . . the awful feelings that I was getting . . . sometimes [I felt] like maybe I just can’t survive’ (p. 15). For some, their experiences impacted their physical health: ‘I had a migraine all the time. I know that’s overload now and migraines are just a part of it’ (p. 02). For others, their mental health suffered: ‘I don’t think I’ve had a career to be honest . . . I get up and keep trying [and] get thrown back down again. I’m very aware it affects my mental health’ (p. 04). One person also reported turning to alcohol to self-medicate: ‘All I got was the crap work, and I hated going, and I was ending up with a drink problem’ (p. 14).

As a result of their poor employment experiences, many participants reported taking career breaks – which ultimately affected their career progression: ‘Every day I would get a little bit more burnt out, and I suddenly crashed in the middle, so I resigned [and then] it was more me overcoming that for about 3 years’ (p. 04). Unfortunately, several participants had not been able to return to work due to the deterioration of their mental health: ‘I left that job and within a couple of years, I would be on disability benefits. And I still am. . .it’s been almost a decade now’ (p. 15). Participants who had returned to work following a career break felt that their sporadic employment had negatively impacted their career success and self-worth: ‘I’m a failure in some ways: I don’t have a secure income, I don’t have savings. That lack of consistency has impacted myself and my family a lot. . .I feel I’ve failed my family’ (p. 02).

## Discussion

We spoke to 18 autistic people about their career journeys and experiences of career success. Our participants’ journeys were marked by a range of experiences that highlighted both challenges and opportunities for positive outcomes. Career paths were often unpredictable, influenced by personal and external factors, including the timing and impact of an autism diagnosis. Success was felt to depend on the presence of adequate and continued career guidance and support, inclusive work environments, and strong workplace connections. While some participants found success by leveraging their skills and successfully requesting workplace adjustments, many faced barriers such as inadequate support, social isolation and negative workplace dynamics. Participants highlighted the crucial role of organisations in creating an inclusive environment that facilitates long-term career success and mitigates the potentially catastrophic effects of poor employment experiences.

Disappointingly, not all our participants felt they had successful careers. Among those who did, their employment outcomes and journeys varied significantly. While some strived for external indicators of success (e.g. promotions, higher salary), others found fulfilment in more intrinsic aspects of their careers, such as personal growth and helping others. This tendency to prioritise intrinsic over external indicators of career success aligns with monotropism theory (D. Murray et al., 2005; F. Murray, 2019), which posits that autistic people’s interests exert a stronger pull than those of other people. Consequently, this monotropic thinking style may lead to greater fulfilment in careers that allow for deep engagement with personal interests and values, rather than those primarily offering conventional markers of success. These findings also emphasise the importance of *career fulfilment* for positive outcomes, as opposed to solely external measures of success, which is consistent with evidence from the general population (e.g. Allan et al., 2019; Duffy et al., 2016; Steger et al., 2012). Next, we consider our findings with reference to extant research and provide recommendations for future research and practice.

Our participants highlighted a pervasive lack of adequate support across their whole career journeys. Where support did exist, participants felt this support focussed too heavily on obtaining entry-level or stereotypically autism-friendly jobs without considering the individual’s match to the role, or the long-term sustainability of the employment (see also Davies et al., 2024). Consequently, our participants highlighted an urgent need for improved, continued support to allow autistic people not just to enter the workplace, but to thrive in the workplace. When designing such support, it will be important to consider that support needs are rarely static, instead evolving over time. Indeed, many of our participants encountered unpredictable life events, such as personal or familial ill-health, which changed their career trajectory and often their own support needs. Evidence from the general population suggests similar impacts of life events, such as changes in caring responsibilities, on career trajectories and evolving support needs (Kossek & Lee, 2017; Maxwell et al., 2019; Millward, 2006). Autistic members of our team therefore emphasised the importance of considering the intersectional nature of people’s multiple identities (e.g. autistic *and* a parent/carer) and subsequent support needs. Organisations may seek to adopt a more holistic approach to support, acknowledging that individuals’ career journeys are necessarily influenced by their broader life circumstances.

Of course, some employees may not feel comfortable disclosing personal details to employers (Lindsay et al., 2019; Romualdez et al., 2021). Thus, regular check-ins could involve open-ended conversations about changing workplace needs, without requiring disclosure of private events, to ensure adjustments can be adapted to match employees’ evolving requirements over time. With employees directing these conversations, they can choose which aspects of their lives, if any, to share while still receiving evolving accommodations. Ultimately, the aim should be flexible, customised ongoing supports that do not depend upon, but also do not preclude, conversations around life circumstances.

Another important aspect of career success for our participants involved social connection within the workplace. Contrary to stereotypes that portray autistic people as wanting to avoid social interactions (e.g. Chevallier et al., 2012), our findings highlighted the crucial role that relationships played in our participants’ professional experiences. For many, fostering positive social connections at work not only nurtured a profound sense of belonging but also formed an integral part of their overall positive work experiences. These findings align with prior research emphasising the importance of positive workplace relationships for autistic employees (Dreaver et al., 2020; Hayward et al., 2019a; Martin et al., 2023).

However, establishing these positive workplace relationships often proved challenging. Neurotypical expectations for workplace socialisation were sometimes perceived as difficult to meet (Bertilsdotter Rosqvist, 2019; Bertilsdotter Rosqvist et al., 2023b). For example, one participant reported feeling uncomfortable with team lunches and often called in sick to avoid them. Absence at such events, however, was misunderstood by colleagues, contributing to perceived relational friction. These experiences exemplify the ‘double empathy problem’ (Milton, 2012), which posits that social challenges stem from a *mutual* lack of understanding between autistic and non-autistic people. To navigate these social situations, several participants reported feeling compelled to mask their autistic traits. While this strategy may temporarily ease social interactions, it can be exhausting and unsustainable in the longer term (Bertilsdotter Rosqvist et al., 2023a; Pearson & Rose, 2021; Pryke-Hobbes et al., 2023). In some cases, the reported mismatch in social expectations and communication styles between autistic and non-autistic colleagues escalated to more serious issues. Several participants faced bullying and exploitation, which they attributed to being perceived as ‘different’ from their colleagues (Djela, 2021; National Autistic Society, 2016; Sreckovic et al., 2024). These negative experiences often came at significant personal and professional costs, with some terminating their employment as a result (Cooper & Kennady, 2021).

More broadly, participants felt that ‘social abilities’ were often a prerequisite for conventional career success, which was perceived to disproportionately disadvantage autistic employees. For example, they said that overreliance on social skills in job descriptions may unintentionally create barriers for autistic people both when entering the workforce, and when applying for higher-level positions. Similarly, our participants suggested that autistic people may find networking events particularly challenging (see also Davies et al., 2024). As a result, autistic people may avoid such social events, which may be misunderstood by their non-autistic counterparts, resulting in less favourable impressions (DeBrabander et al., 2019; Sasson et al., 2017; Sasson & Morrison, 2019). Even where autistic people are able to attend networking events, the relative ‘costs’ of attendance are likely to be greater than for non-autistic people who may not feel as much pressure to mask to fit in or succeed (Pryke-Hobbes et al., 2023). Moreover, the reliance on networking as a pathway to career success may doubly disadvantage autistic people who bear additional responsibilities. For example, evidence in the general population suggests that employees with caring responsibilities have less time to network outside of work and are less likely to engage in ‘informal networking’, due to time constraints (Henderson et al., 2018; Xu & Martin, 2011). Consequently, autistic people with such additional responsibilities may face compounded challenges in achieving objective measures of career success, such as promotion.

It is important to note, however, that while some of our participants aspired to achieve objective measures of career success, others recounted having no interest in such linear progression, especially when such progression involved an increase in managerial responsibilities. Indeed, many expressed a desire for fulfilling careers without the pressure to assume roles that did not align with their preferences or strengths. These findings are in line with evidence from the general workforce, suggesting that not all employees necessarily seek linear career progression and/or managerial responsibilities (Gerber et al., 2009; Hirschi & Koen, 2021). We must therefore be cautious about over-pathologising the career aspirations of autistic people: just as in the broader workforce, autistic people have diverse career goals and should feel they can define their own paths to success. Nonetheless, where autistic people do desire more objective forms of career success, they should be supported to do so. This may include providing practical support for autistic people where networking is required. Potential suggestions in this regard include the use of coloured lanyards to indicate where somebody feels able to initiate conversation, or prefers no physical touch and so on, as well as cards containing potential talking points to provide more structure. Such adjustments may help level the playing field, ensuring that autistic people have equal opportunities to advance their careers in a way that suits their needs and preferences.

Our participants also highlighted the important role organisations play in ensuring the career success of autistic people. Organisational inclusion and accessibility were considered essential for the long-term sustainability of autistic employees’ careers, yet most workplaces were perceived by our participants as falling short of meeting these needs. As a result of their unsustainable employment experiences, some participants found themselves compelled to work part-time (Davies et al., 2022). However, such adjustments often came at a significant personal cost, such as the loss of income. For others, self-employment seemed like the only viable option, offering greater control and the ability to tailor a work environment to suit their individual needs. Nonetheless, self-employment posed distinct challenges, including the need to market oneself and the potential for subsequent financial instability (Kelly & Romualdez, 2022). Additional support for autistic freelancers and entrepreneurs may therefore be warranted. One potential avenue for providing such support could be through grants that are designed to help autistic people develop effective marketing strategies. Grant funding could, for example, be spent on attending training programmes, utilising mentorship initiatives, or be devoted to constructing robust personal and professional brands.

However, self-employment should not be considered an adequate solution for non-inclusive and inaccessible workforces, and autistic people should not feel self-employment is the only viable option. Employers must work towards supporting autistic talent within their organisations. To that end, employers may benefit from tailored guidance (e.g. training, resources) aimed at supporting autistic employees already within their organisation. While several autism-specific training programmes have been developed and evaluated, few have been conducted in employment contexts, and those that have tend to focus on outcomes such as gains in knowledge about autism, as opposed to tangible improvements to support (see Ashworth et al., 2023, for an overview). Nonetheless, one recent study examined the impact of an online autism training programme for employers on their commitment to inclusion and found that only a minority of participants (only 7 of 120, 5.8%) showed a significant increase in their commitment to inclusion (Ashworth et al., 2023). These findings suggest that training alone may not be sufficient for improving autistic people’s employment experiences, and thus the sustainability of employment for autistic people. Future research must involve autistic people and employers working together to develop and evaluate initiatives that genuinely improve experiences, allowing autistic people to thrive in the workplace.

### Limitations

This study is not without its limitations. First, although we did attempt to diversify our sample, including purposively recruiting minority-ethnic participants, most of our participants were well-educated, and almost half of those currently employed worked in the education sector (*n* = 6, 46.2%). It is therefore important to recognise that our findings reflect the experiences of a relatively narrow group of autistic people. Future research should prioritise the inclusion of autistic people with varied educational backgrounds, particularly those with less formal education, given that education and experience can play a pivotal role in experiences of career success (Davies et al., 2024). Second, most of our participants (*n* = 8, 61.5%) did not hold any managerial responsibilities. Hearing from more autistic people in higher positions, particularly those with managerial roles, may provide a well-rounded perspective on career success and help to identify factors associated with such success. Finally, over half of our participants (*n* = 10, 55.6%) reported having worked for seven or more employers, suggesting a tendency for job transitions. This experience may have negatively skewed perceptions of career progression and success, as participants may not have had time to progress within one organisation. Alternatively, it may be that such job transitions are a common experience among autistic people. Future research should examine the dynamics of job transitions, and how such transitions impact career success and satisfaction within the autistic community.

## Conclusion

This study highlights the diverse and often challenging career journeys of autistic people. Supporting autistic people to achieve career success and fulfilment requires taking a holistic, lifelong approach rather than just focusing on entry-level hiring. Fostering truly inclusive and accessible workplaces is crucial, alongside providing tailored support that evolves across individuals’ employment journeys. Only through collaborative efforts between autistic people, support services, employers and policymakers can we enable autistic people to flourish at work.

## Appendix 1

### Work history interview guide

Hi, my name is [Interviewer], and I’m a researcher on this project. I’m also autistic. [Interviewer] is also here who will be recording the interview, if that is okay with you. She will keep her microphone and camera off during the interview.

In this project, we’re recording the work histories of autistic people. We want to understand what people’s work experiences have been like, including things like the support they have had, and how they have progressed to where they are now.

We’re very thankful that you’ve chosen to meet with us and share a bit about your employment history. This interview is taking place because you’ve agreed to share parts of your story, and you can stop or take a break any time.

If you do want to take a break, just let us know, either in the chat box, by telling me verbally or by putting your hand up.

I’m going to ask you to talk through your career timeline that you created. I will also ask some general questions about your employment history. Sometimes I’ll ask you to explain what you’ve said in more detail to get a clearer idea of the things you’re telling me. Just answer at your own pace, there’s no rush.

You can skip a question or come back to a question at any time. Please ask me to repeat or re-word a question if you need to.

Do you have any questions, before we start?

Can I just double check that you are happy for our conversation to be recorded? Only the audio will be saved. This will just help us remember exactly what we discussed today!

*[Interviewer] to start recording*

––-

Okay, so we are going to start the interview now. First, I will ask you some questions about your background and about your education. You can use your timeline as a prompt if this is helpful. Once I have asked these questions, I will ask you to talk me through your timeline. I will stop you every now and again to ask you some more specific questions.

We will start with some questions about your background if that is okay. This is just so we know a bit more about the different kinds of people we are speaking to, and the context behind their experiences.

Before we get started, can I ask when you received your autism diagnosis, or when you first identified as autistic?

#### Question 1: to start, can you tell me about your teenage years? For example, who you lived with, and what your education was like

*Potential prompts (not all will necessarily be relevant or asked in the interview)*:

(a) What decade? Did growing up in this decade have any impact on the types of job opportunities you had? Did this have any impact on your perspective about work?
(b) Where in the UK did you live? Did this have any impact on the types of job opportunities you had? Did this have any impact on your perspective about work?
(c) Who was in your family? Who did you live with? What were these relationships like?
(d) Did your parents/carers work? What did they do? Was your family comfortable financially?
(e) What kind of input (if any) did your family have on your career planning during your teenage years?
(f) What kind of role models did you have? Who did you look up to?
(g) Was education important to you/your family? Was work important to you/your family?
(h) What kind of secondary school did you go to?
(i) What was your experience of secondary school? Positive/negative/mixed?
(j) Did you feel supported during your teenage years? Why/why not?
(k) Did you finish school? If not, why not?
(l) Did you get any qualifications when you finished secondary school? If so, how did you feel about your grades?
(m) What were your aspirations during this time? What did you want to do in the future? Did you have a plan?

#### Question 2: I’d like to discuss more about what you did after secondary school now

From your timeline, I can see that you [*went to college/sixth-form/apprenticeship/work*]. Can you tell me a bit more about that?

*Potential prompts (not all will necessarily be relevant or asked in the interview)*:

(a) What made you choose this route?
(b) What was going on in your life at this time? How did you feel?
(c) What was your family life like? Did this have any impact on your career/education choices?
(d) How would you describe your experience? Positive/negative/mixed?
(e) Who were your role models during this time? Who did you look up to?
(f) Did you feel supported? Why/why not?
(g) Did you get any qualifications? If so, how did you feel about your grades?
(h) What were your aspirations? What did you want to do in the future?
(i) Did you get any career advice (e.g. from job coaches/career advisors/family/friends)?
(j) What were your aspirations during this time? What did you want to do in the future? Did you have a plan?
(k) How did you feel about your future? Optimistic/pessimistic/mixed?

#### Question 3: I’d like to move on now to your career timeline

Please can you talk me through your timeline from the start to the end?

*Potential prompts (not all will necessarily be relevant or asked in the interview)*:

(a) Further education/training – why? How would you describe your experience? Positive/negative/mixed?
(b) How was your first job?
(c) How did you know it was time to move jobs? What factors did you consider (e.g. time/salary/responsibility/personal circumstance)?
(d) Did you disclose to any employers? How did this impact your career?
(e) What made you stay in a job for a long period? (e.g. salary/security/sameness)
(f) How did you feel when you were transitioning from one job/employer/role to another? Why? Could this have been improved?
(g) Job-matching?
(h) Relationships with colleagues? Positive/negative/mixed? Did this impact your career?
(i) Notable absences – why? How did you feel going back to work? Did this impact your career?
(j) Having children/caring responsibilities – how did this impact your career?
(k) Have you had any career support (e.g. career advisors/job coaches)? How was this organised? How did you find it? Positive/negative/mixed?
(l) Have you felt supported during your career? Why/why not?

#### Question 4: thanks so much for talking through your timeline

What I’d like to do now is ask a few questions about your career overall. So, thinking about your career as a whole, are you happy with how it has gone?

*Potential prompts (not all will necessarily be relevant or asked in the interview)*:

(a) Why/why not?
(b) Has your career gone how you expected? Why/why not?
(c) Who are your role models? Who do you look up to?
(d) Do you think being autistic has impacted your career at all? How? Has anything else impacted your career?
(e) What could have improved your experience?
(f) What are your career goals for the future? How are you planning to achieve these goals?

#### Question 4: almost done! Finally, I wanted to ask some more specific questions about career progression

You might have already answered some of them during our discussion of your career timeline. But I’ll ask them anyway just in case you have any further reflections. To start, what does that term–career progression–mean to you?

*Potential prompts (not all will necessarily be relevant or asked in the interview)*:

(a) Is career progression important to you? Why/why not?
(b) How do you measure career progression?
(c) Are you happy with how much you have progressed in your career? What could have been done differently?
(d) What do you think has impacted your career progression? (e.g. personal factors, organisational factors)
(e) Tips for employers?
(f) Tips for other autistic people?

**Next steps**

That’s all my questions. Thanks so much for speaking with me and for sharing your work history – it was wonderful to be able to hear it. I hope that you are feeling ok about it too.

**Do you have any questions, or anything to add before [Interviewer] turns the recording off?**

*[Interviewer] turns the recording off*

Great. Regarding next steps, [Interviewer] is going to check over the recording of the interview and make sure the transcript is accurate. She will send you the transcript (the written-out version) of your interview within the next 2 weeks for you to look over and make sure that you are happy with everything being included.

I hope that you enjoyed our conversation. But if you feel worried or upset as a result of what we’ve spoken about today, please do reach out for support. [Interviewer] included the contact details for the mental health charities Mind, the Samaritans, and SHOUT in the information sheet that you read at the start of this project and will re-send them to your email today. She will also send over your £20 voucher.

Thanks so much again and we look forward to being in touch again soon.

## Notes

1. While all participants were current residents of the United Kingdom, five participants (27.8%) had experienced some education and/or employment in other countries.

2. At the time of data collection, the median salary in the United Kingdom was £27,716 (Office for National Statistics, 2022).
